# Old Blood, Young Bones: Identification of Middle‐Aged Myeloid Cells That Limit Cortical Bone Loss

**DOI:** 10.1111/jcmm.71094

**Published:** 2026-03-23

**Authors:** Jinsha Koroth, Ismael Y. Karkache, Elizabeth K. Vu, Joseph C. Manser, Mitchel J. Shimak, Kim C. Mansky, Elizabeth W. Bradley

**Affiliations:** ^1^ Department of Orthopedic Surgery, Medical School University of Minnesota Minnesota USA; ^2^ College of Veterinary Sciences University of Minnesota Minnesota USA; ^3^ Division of Orthodontics, School of Dentistry University of Minnesota Minnesota USA; ^4^ Stem Cell Institute University of Minnesota Minnesota USA

**Keywords:** bone, bone remodeling, osteoblast, osteoclast, osteoclast progenitor, osteomac, osteoprogenitor

## Abstract

Although studies support disrupted bone remodelling within geriatric populations, mid‐life changes are understudied. To investigate this, we performed bone marrow transplantation assays using either 8‐ or 40‐week‐old mice. Micro‐CT analyses of lethally irradiated 8‐week‐old mice transplanted with 40‐week‐old bone marrow exhibited increased mid‐shaft femoral cortical bone mass and thickness. Intensive bone marrow regeneration mirrors hematopoietic development in that erythro‐myeloid progenitors (EMPs) first expand to support blood production before definitive hematopoietic stem cell (HSC) production. We hypothesized that reduced HSC capacity of 40‐week‐old bone marrow and compensatory expansion of EMPs may facilitate gains in cortical bone. Flow cytometry analyses revealed greater EMP to HSC ratios when mice were reconstituted with increasing percentages of middle‐aged bone marrow. To identify cell types mediating these effects, we performed comparative scRNA‐Seq analyses and identified CD11B^+^CD36^+^ myeloid cells exhibiting enriched expression of bone anabolic cytokines. Elevated levels of Wnt ligands, especially Wnt6, characterized these cells. In lineage tracing assays, CD11B^+^CD36^+^ cells were donor‐derived myeloid cells. In functional assays, we demonstrate that soluble factors produced by CD11B^+^CD36^+^ cells enhance osteogenesis. Moreover, CD11B/CD36/Wnt6 exquisitely mark anabolic macrophages within human bone marrow. These findings reveal a myeloid population present during midlife that enhances cortical bone.

## 1 Introduction

Bone mass undergoes significant changes across the human lifespan, with a well‐recognized pattern of attainment of peak bone mass around the third decade of life. While onset of clinically defined osteoporosis or osteopenia typically occurs later in life (e.g., 50 for women and 70 for men), bone loss begins as early as the third decade of life. Understanding the physiological changes to the skeleton during midlife and the mechanisms by which bone mass is maintained during this period may therefore help to limit the onset of osteoporosis.

Remodelling of bone occurs throughout the lifespan and is facilitated through the coordinated actions of bone resorption, mediated by osteoclasts, and bone formation facilitated by osteoblasts. Increasing age tips the balance between bone formation and resorption, leading to a net loss of bone mass. While critical control of osteoblast and osteoclast numbers and activity primarily governs bone mass, other cells such as macrophages help to modulate the activity of these cells; thus, examining how different types of macrophages impact bone mass may provide additional avenues for therapeutic development.

Bone marrow resident macrophages include both osteoclasts and bone anabolic macrophages, known as osteomacs, amongst others. Osteoclasts that help to model the skeletal elements during development derive from erythromyeloid progenitors (EMPs) [1, 2, 3]. In contrast, osteoclasts that facilitate bone remodelling later in life trace their origins to hematopoietic stem cells (HSCs) [1, 2]. Evidence supporting fusion between EMP‐ and HSC‐derived cells is likewise found in murine model systems [1, 2]. In addition to regulating HSC activity [4], osteomacs also interact with osteoclasts and osteoblasts to regulate bone resorption and formation, respectively [5, 6]. The developmental origins of osteomacs have not been conclusively defined.

EMPs and HSCs both contribute to the development of the hematopoietic system, which progresses through distinct waves, each contributing to the establishment of monocyte/macrophage lineage cells in the body. The first wave generates early EMPs in the foetal yolk sac around embryonic day E7.5 [7]. At E8.25, a second wave of EMP generation occurs [7]. These phases of primitive hematopoiesis lead to the formation of tissue‐resident macrophages that exhibit a strong capacity for self‐renewal [8]. HSCs emerge later, around E10.5, colonize the bone marrow, and give rise to circulating monocyte‐derived macrophages [9]. Recapitulation of this process also occurs during intensive hematopoietic regeneration in adults, where reconstitution of HSCs occurs in conjunction with erythro‐myeloid progenitor activity to facilitate blood production [10, 11]. Consequently, both EMP‐ and HSC‐derived monocyte–macrophage lineage cells are present within the post‐natal bone marrow niche where they both influence the bone remodelling process [12]. Likewise, macrophages within the spleen can be recruited to facilitate bone regeneration [2].

Recent studies have suggested that reactivation of developmental programs during adulthood can rejuvenate cells and organ systems. For instance, partial or temporary induction of cellular reprogramming factors, known as Yamanaka factors, can rejuvenate somatic cells and tissues without loss of their differentiated state [13, 14, 15]. However, integration with classical developmental signalling pathway reactivation (e.g., Wnts, Notch) remains underexplored. Likewise, the effects of induction of developmental programs during midlife are unknown.

Recent evidence suggests that aging of biological systems is not uniform overtime but rather characterized by several punctuated periods of accelerated aging [16]. These periods of greater rates of aging associate with significant molecular alterations occurring around the ages of 44 and 60 in humans [17]. Given this, understanding the biological changes that occur during midlife (e.g., 40–65 years of age) may prove vital for optimizing aging, identifying potential issues, and promoting interventions to improve quality of life; yet, there is a gap in our understanding of the aging process between adult development and age‐related decline [18]. The purpose of this study is to identify cellular and molecular mechanisms that maintain the skeleton during midlife. We hypothesized that reactivation of developmental programming facilitating primitive hematopoiesis during intensive bone marrow regeneration could have a positive effect on the skeleton.

## 2 Methods

### 2.1 Animal Procurement and Housing

The Jackson Laboratory provided male C57Bl/6J mice (strain #000664, Bar Harbour, ME) aged to either 4–6 or 38 weeks old. Mice were then acclimated for two weeks before study initiation. Forty‐week‐old mice were chosen to reflect middle age [19]. A subset of 8‐week‐old male B6.SJK‐*Ptprc* ^a^Pepc^b^/BoyJ (*n* = 4) were also purchased for use in transplantation studies. B6.Cg‐*Gt(ROSA)26Sor* ^*tm14(CAG‐tdTomato)Hze*^/J (strain #:007914), referred to as Ai14 tdT, were purchased from the Jackson Laboratory and mated with mice harbouring the LysM‐Cre allele (Jackson Laboratory). All animal research was conducted according to guidelines provided by the NIH and the Institute of Laboratory Animal Resources, National Research Council. The University of Minnesota IACUC approved all animal studies.

### 2.2 Whole Bone Marrow T Cell Depletion

Male 6–8‐ or 40‐week‐old C57BL/6J mice were euthanized by CO_2_ asphyxiation, femora and tibiae were collected, and the marrow cavity was flushed with sterile PBS. Whole bone marrow suspensions from age‐matched mice were pooled, pelleted, and then suspended in 1× RBC lysis buffer (#00–4333‐57, ThermoFisher Scientific, Waltham, MA). Cells were pelleted and suspended in MACS buffer. Cell suspensions were then incubated with PE‐conjugated anti‐CD8 and anti‐CD3 antibodies, washed, pelleted, and suspended in MACS buffer. Cells were then incubated with anti‐PE microbeads (Miltenyi Biotec, #130–048‐801), washed, and then applied to an LS column (#130–042‐401, Miltenyi Biotec) placed in a MidiMACS Separator magnet. Eluted CD8^−^/CD3^−^ bone marrow cells were suspended in PBS.

### 2.3 Irradiation and Bone Marrow Reconstitution

Six to 8‐week‐old male mice (*n* = 18) were lethally irradiated using two 500 rad doses separated by a period of 4 h, and then randomly transplanted with T cell‐depleted bone marrow derived from either age‐matched males (*n* = 10) or 40‐week‐old males (*n* = 8). Following bone marrow transplantation, mice were housed in autoclaved cages and administered polymyxin B and neomycin sulphate for two weeks. Mice were aged to 24‐weeks‐old and then euthanized for analyses. All animals were included within the study.

### 2.4 Micro‐Computed Tomography

Right femora collected from 24‐week‐old chimeric mice and non‐irradiated 24‐week‐old males were fixed in 10% neutral buffered formalin for 48 h and stored in 70% ethanol. Blinded scanning was performed using the XT H 225 micro‐computed tomography (micro‐CT) machine (Nikon Metrology Inc., Brighton, MI) set to 120 kV, 61 μA, 720 projections at two frames per projection with an integration time of 708 milliseconds as previously described [20]. Scans were done at an isometric voxel size of 7.11 μm with a 1 mm aluminium filter. Each scan volume was reconstructed using CT Pro 3D (Nikon Metrology Inc., Brighton, MI), and then converted to bitmap datasets using VGStudio MAX 3.2 (Volume Graphics GmbH, Heidelberg, Germany). Scans were reoriented via DataViewer (SkyScan, Bruker micro‐CT, Belgium) to create a new bitmap dataset. Morphometric analysis was performed using SkyScan CT‐Analyser (CTAn, Bruker micro‐CT). 3D Analysis of trabecular bone was performed in the distal metaphysis 0.7 mm proximal to the growth plate and extending 1.5 mm proximally toward the bone diaphysis. For the bone cortex, 2D analysis occurred in a 0.5 mm section within the mid‐diaphysis defined as 4 mm from the growth plate. Regions of interest were set by automated contouring for the trabecular and cortical ranges, with some manual editing when necessary. 3D models of the ROIs were reconstructed using CT Vox for qualitative analysis of bone.

### 2.5 Bone Histomorphometry

Tibiae from chimeric mice were fixed in 10% neutral buffered formalin then decalcified in 15% EDTA for 14 days. Tibiae were paraffin embedded, 7‐μm sections; longitudinal sections were generated. Sections were then TRAP/Fast Green stained (Sigma‐Aldrich, St. Louis, MO, USA; #387A‐1KT) or Masson's trichrome stained (Sigma‐Aldrich, #HT15‐1KT). Standardized histomorphometry was performed to assess osteoclast and osteoblast number per bone surface as well as osteoclast perimeter per bone perimeter [21].

### 2.6 Collection of Bone Marrow

Commercially available human bone marrow from a 45‐year‐old female was procured (Lonza, #1 M‐105). Collection of human bone marrow aspirates was approved by the Salus Institutional Review Board for Lonza Inc. (Protocol Salus Number 00074). Written informed consent was obtained from all individual participants included in the study. All donors were screened for normal blood cell counts and infection (e.g., CMV, HIV, Hepatitis B/C) before collection of bone marrow aspirates. Subjects were informed of the procedural risks, including infection, bleeding, and pain, and provided signed, written informed consent before marrow collection. Bone marrow aspirates were collected from the iliac crest under local anaesthesia under standard procedures. The tissue was collected within 24 h of shipment, transported at ambient temperature, and processed upon arrival. RBCs from human bone marrow aspirates were lysed and nucleated cells were used to sort for CD11B^+^ cells as described subsequently.

### 2.7 Magnetic Assisted Cell Sorting

Bone marrow cells were flushed from the femora and tibiae of 24‐week‐old chimeric mice from each group or from 40‐week‐old male and female C57Bl6/J mice and red blood cells were lysed. Cells were pelleted and suspended in MACS buffer. Nucleated cells were then incubated with CD11B microbeads (Miltenyi Biotec, #130–126‐725). Cell suspensions were then applied to an LS column placed in a MidiMACS Separator magnet, washed three times with MACS buffer, and eluted from the column. For isolation of CB11BCD36^+^ cells, cells were incubated with Allophycocyanin (APC)‐conjugated anti‐CD36 (Miltenyi Biotec, #130–122‐085) antibodies. Cells were pelleted, washed, suspended in 1× MACS buffer and incubated with anti‐APC microbeads (Miltenyi Biotec, #130–090‐855). Cells were washed and then suspended in MACS buffer and separated using an LS MACS separation column placed within the magnetic field of a MACS Separator magnet. Unbound cells were used to isolate CD11B^+^CD36^−^ cells. Eluted cells were then pelleted and the supernatant was removed and discarded. To isolate CD11B^+^ cells, cells were then suspended in 1× MACS Buffer and incubated with phycoerythrin (PE)‐conjugated anti‐CD11B (Miltenyi Biotec, #130–113‐235). Cells were washed, suspended in MACS buffer, and incubated with anti‐PE microbeads (Miltenyi Biotec, #130–048‐801). Magnetic sorting for PE‐labelled cells was accomplished as described above.

### 2.8 Flow Cytometry

Flow cytometry analysis for HSC and EMP analysis was performed using BD LSRFortessa X‐20 flowcytometer and data were analysed with BD FACSDiva and FlowJo software. A 12 colour antibody panel was designed using Fluorofinder software and the screening panel is shown in Table 1. The bone marrow cells were isolated as previously described, stained with the indicated antibodies for 20 min on ice and washed with PBS to remove unbound, excess antibodies. Single stained controls prepared using UltraComp eBeads Plus Compensation Beads (ThermoFisher, 01–3333–41) were used for compensation, and fluorescence minus one (FMOs) controls were used to define the gating region. Sytox Green was used to exclude dead cells before analysis. At least 30,000 events were acquired per sample. For flow cytometry analysis of CD11B^+^CD36^+^ cells, we prepared single stained controls and double stained samples (*n* = 3) with unsorted nucleated cells with PE‐conjugated anti‐CD11B, APC‐conjugated anti‐CD36, or co‐stained with anti‐CD11B and anti‐CD36 antibodies, respectively. For assessment of chimerism, we prepared single stained controls and double stained samples (*n* = 3) with unsorted nucleated cells with BV421‐conjugated anti‐CD45.1 (Biolegend, #110731), APC‐conjugated anti‐CD45.2 (Miltenyi Biotech, #130–102‐964), or co‐stained with anti‐CD45.1 and anti‐CD45.2 antibodies, respectively. Data were acquired on a Fortessa X‐20 and analyses were performed using BD FACSDiva and FlowJo software.

**TABLE 1:** Antibodies used for flow cytometry.

Marker	Colour/Format	Company	Catalogue	Laser/Ex Filter	Em Filter
Lineage Cocktail (CD3/Ly‐6G/CD11b/CD45R/Ter‐119)	Alexa Fluor 700	BioLegend	133,313	640	730/45710LP
CD117	APC‐eFluor 780	Thermo Fisher Scientific	47–1172‐80	640	780/60750LP
CD41	Brilliant Violet 786	BD Biosciences	740,903	405	780/60750LP
CD16/32	Brilliant Violet 711	BioLegend	101,337	405	710/50690LP
CD45	Brilliant Violet 510	BioLegend	103,137	405	525/50505LP
CD49f	PE‐Cy7	BioLegend	313,621	561	780/60750LP
CD90	APC	Thermo Fisher Scientific	A14727	640	670/30
CD45RA	Brilliant Ultraviolet 395	BD Biosciences	740,232	355	379/28
CD38	Brilliant Ultraviolet 805	BD Biosciences	741,955	355	785/62750LP
CD34	Brilliant Violet 421	BioLegend	152,207	405	450/50
Impermeant NA	Sytox Green	Thermo Fisher Scientific	S7020	488	525/50505LP
Fluorescent Protein	tdTomato			561	586/15

### 2.9 Single Cell RNA‐Sequencing

CD11B^+^ cells from chimeric mice in each group or 40‐week‐old male C57Bl/6J mice were isolated via MACS as described above. CD11B^+^ cells were likewise collected from human bone marrow aspirates. Single cell cDNA libraries from each sample were then prepared using Chromium Single cell 3′ Reagent v3 Kits (10× Genomics, Pleasanton, CA, USA) according to the manufacturer's protocol as we have previously described [22]. Quality of amplified cDNA was assessed using an Agilent Bioanalyzer. Following quality control, the cDNA libraries were sequenced on an Illumina NovaSeq 6000 sequencer at a depth of 50,000 reads per cell. The Genomics Core at the University of Minnesota performed library construction and sequencing.

### 2.10 Single Cell Bioinformatic Analyses

10× Genomics Cell Ranger software (version 6.0.0) was used to process the raw scRNA‐seq data in accordance with the guidelines specified by the manufacturer. Cell Ranger software (2020‐A, 10× Genomics Cell Ranger Count v7.0.1) was used to demultiplex the raw base call (BCL) files produced by the Illumina NovaSeq 6000 sequencer. Next, Cell Ranger was used to count the unique molecular identifiers (UMI) and barcodes and align fastq files to the mouse or human reference genomes (GRCm39 or GRChg38, respectively). Quality assurance and subsequent studies were then performed on the feature‐barcode matrices created by Cell Ranger. The study was filtered to exclude cells with outlier status, aberrant gene detection rates (< 500 and > 5000), and high mitochondrial transcript level (> 8%, a sign of cellular stress). Then, using uniform manifold approximation and projection (UMAP) or t‐distributed stochastic neighbour embedding (t‐SNE), a non‐linear dimensional reduction was carried out, enabling for the identification and visualization of different cell clusters. Data integration was performed using Cell ranger to compare between samples and analysed using Cloupe Browser (10× Genomics).

### 2.11 Modified Lineage Tracing

Recipient 8‐week‐old CD45.1^+^ mice were lethally irradiated and transplanted with T cell‐depleted bone marrow from Ai14 tdT:LysM‐Cre CD45.2^+^ mice. Four weeks after transplantation, we collected the bone marrow from chimeric mice and performed flow cytometry for CD11B/CD36 and assessed the percentage of tdT^+^ cells.

### 2.12 Culture of CD36 ^+^ CD11B ^+^ Cells

CD11B^+^CD36^−^ and CD36^+^CD11B^+^ cells were isolated via MACS and cultured at a density of 0.26 × 10^6^ per cm^2^ in α‐Minimal Essential Medium supplemented with 50 ng/mL recombinant mouse macrophage‐colony stimulating factor (M‐CSF) (#416‐ML, R&D Systems, Minneapolis, MN). Cells were maintained in culture and supplemented with M‐CSF for 7 days or until adherent. Conditioned medium was then collected, filtered with a 70 μm filter, and stored at −80C. Cells were lysed in TriZol reagent (ThermoFisher Scientific, #15596018) for gene expression analyses. For osteoclastogenesis experiments, cells from each fraction were cultured in αMEM supplemented with 100 ng/mL RANKL (R&D Systems, #462‐TR‐010) and 50 ng/mL M‐CSF as previously described [23] and TRAP stained using the Leukocyte Acid Phosphatase (TRAP) kit (Sigma‐Aldrich, #387A‐1KT).

### 2.13 RNA Isolation and Semi‐Quantitative RT‐PCR

Total RNA was extracted using TRIzol and chloroform. Reverse transcription using the iScript Reverse Transcription Supermix (Biorad, #1708841) with 1 μg of total RNA was used to generate cDNAs. Gene expression was then assessed via real‐time PCR using gene‐specific primers listed in Table 2. Fold changes in gene expression for each sample were calculated using the 2^−ΔΔCt^ method relative to control after normalization of gene‐specific C_t_ values to Ywhaz C_t_ values [23, 24]. Shown are averaged data from three independent replicate experiments derived from cells isolated from female animals.

**TABLE 2:** Primers used for qPCR assays.

Gene	Forward Primer	Reverse Primer
*Adgre1* (F4/80)	5′‐CTCAAGGACACGAGGTTGCT‐3′	5′‐GACGGTTGAGCAGACAGTGA‐3′
*Bgn*	5‐‘GAGACCCTGAATGAACTCCACC‐3′3′	5′‐CTCCCGTTCTCGATCATCCTG‐3′3′
*Bmp1*	5′‐TGGCCATATCCAGTCTCCCA‐3′3′	5′‐TGTCGTGACGCTCAATCTCA‐3′3′
*Bmp2*	5′‐CTGCGGTCTCCTAAAGGTCG‐3′3′	5′‐ATGCTGAGCAGCCTCAACTC‐3′3′
*Cd45*	5′‐TGGATTTGCCCTTCTGGACA‐3′3′	5′‐CCAAACATGGCAGCATCACT‐3′
*Cd68*	5′‐TGACCTGCTCTCTCTAAGGCT‐3′	5′‐GTTGCAAGAGAAACATGGCCC‐3′
*Cd86*	5′‐CAGCACGGACTTGAACAACC‐3′	5′‐CTCCACGGAAACAGCATCTGA‐3′
*Cd166*	5′‐GGGAGCGTCATAAACCAAACAG‐3′	5′‐TCTGTTTTCATTCGCAGAGACA‐3′
*Csf1r*	5′‐ACTCTCCAACCTGCATCGGCT‐3′	5′‐GTCCACAGCGGTGAGACTGAG‐3′
*Cx3cr1*	5′‐CCATCTGCTCAGGACCTCAC‐3′	5′‐CACCAGACCGAACGTGAAGA‐3′
*Cd74*	5′‐GGCTCCACCTAAAGAGCCAC‐3′	5′‐GGGTGTGACATCAGGGAACA‐3′
*Kit*	5′‐TCACCAGTGGACGTACAGGT‐3′	5′‐GGGCCTGGATTTGCTCTTTGT‐3′
*Mmp13*	5′‐GCACCCTCAGCAGGTTGAGGC‐3′	5′‐TGAACCGCAGCGCTCAGTCTC‐3′
*Mrc1*	5′‐TTCAGCTATTGGACGCGAGG‐3′	5′‐GAATCTGACACCCAGCGGAA‐3′
*Phospho1*	5′‐ATGAGCGGGTGTTTTCCAG‐3′	5′‐TGCCGTCCCTAGATAGGCATC‐3′
*Pu.1*	5′‐CCTCACCGCCCCTCCAT‐3′	5′‐CACACTCTGCAGCTCTGTGA‐3′
*Siglec1*	5′‐GGCCTATCACACCTGGAGAAAG‐3′	5′‐GTCAGAGAGCAGCAACCACT‐3′
*Sp7*	5′‐GGAGGTTTCACTCCATTCCA‐3′	5′‐TAGAAGGAGCAGGGGACAGA‐3′
*Tnfsf11 (RANKL)*	5′‐GCTGGGACCTGCAAATAAGT‐3′	5′‐TTGCACAGAAAACATTACACCTG‐3′
*Tnfrsf11a (RANK)*	5′‐TAAAGTCTGTGATGCAGGCAAG‐3′	5′‐CCGTATCCTTGTTGAGCTGC‐3′
*Wnt3a*	5′‐TGTTCTGGACAAAGCCACCC‐3′	5′‐AATGTCCTCACTACAGCCGC‐3′
*Wnt6*	5′‐ACTGGGGGTTCGAGAATGTC‐3′	5′‐TCTCTCGGATGTCCTGCTGC‐3′
*Wnt10b*	5′‐ACGCCGGGGAAGCGG‐3′	5′‐AGTCAGTCAGCGCCTCCAG‐3′
*Ywhaz*	5′‐GAGCTGAGCTGTCGAATGAG‐3′	5′‐GATGACCTACGGGCTCCTAC‐3′

### 2.14 Measurements of Proliferation and Osteoblastogenesis

ST2 cells were cultured at a density of 0.015 × 10^6^ cells/cm2 in conditioned medium from CD11B^+^CD36^−^ or CD11B^+^CD36^+^ cells. MSC proliferation was then determined using a Cck8 assay (Cell Counting Kit, Sigma Aldrich, #96992‐100TESTS‐F). To assess MSC migration, transwell assays were performed using Corning Costar transwell assay cell culture inserts (Sigma Aldrich, #CLS3464‐12EA). A total of 2 × 10^4^ cells were seeded into the upper chamber, the lower chamber was filled with an equal mixture of conditioned medium and MEM (10% FBS), and the plate was incubated for 18 h. Following incubation, the insert was washed with 1× PBS two times and non‐migrated cells were carefully removed. Migrated cells were fixed with 100% methanol and Giemsa stained. Dried membranes were mounted on slides using VECTASHIELD Antifade Mounting Medium with DAPI. Cells were visualized and five random fields per insert were counted using Image J.

### 2.15 Western Blotting

Cell lysates were collected in a buffered SDS solution (0.1% glycerol, 0.01% SDS, 0.1 m Tris, pH 6.8) on ice. Total protein concentrations were obtained with the Bio‐Rad D_C_ assay (Bio‐Rad). Proteins (20 μg) were then resolved by SDS‐PAGE and transferred to a polyvinylidene difluoride membrane. Western blotting was performed with antibodies (1:1000 dilution) for WNT6 (Thermo Fisher Scientific, #24201–1‐AP), Active β‐catenin (Millipore, #05–665‐25UG), β‐catenin (BD Biosciences, #610153), phospho‐JNK (Cell Signalling Technology, #4668 T), JNK (Cell Signalling Technology, #9252 T), Histone 3 (Millipore, #05–928), and corresponding secondary antibodies conjugated to horseradish peroxidase (HRP) (Cell Signalling Technology). Antibody binding was detected with the Supersignal West Femto Chemiluminescent Substrate (Pierce Biotechnology, Rockford, IL). Shown are data averaged from three males, but studies were also performed in females (*n* = 3 per group) in three independent replicate experiments, each containing six pooled replicate wells per group.

### 2.16 Statistics

Statistics were performed in GraphPad Prism (Version 10) using Student's *t*‐test or one‐way ANOVA as appropriate and post hoc tests for multiple comparisons when necessary. Specific *p* values are shown within figures, with *p* < 0.05 considered as significant. Data are shown as box plots from the 25th to 75th percentiles, with whiskers extending to the minimum and maximum value and means shown by horizontal lines or means ± standard deviation.

## 3 Results

**FIGURE 1:**
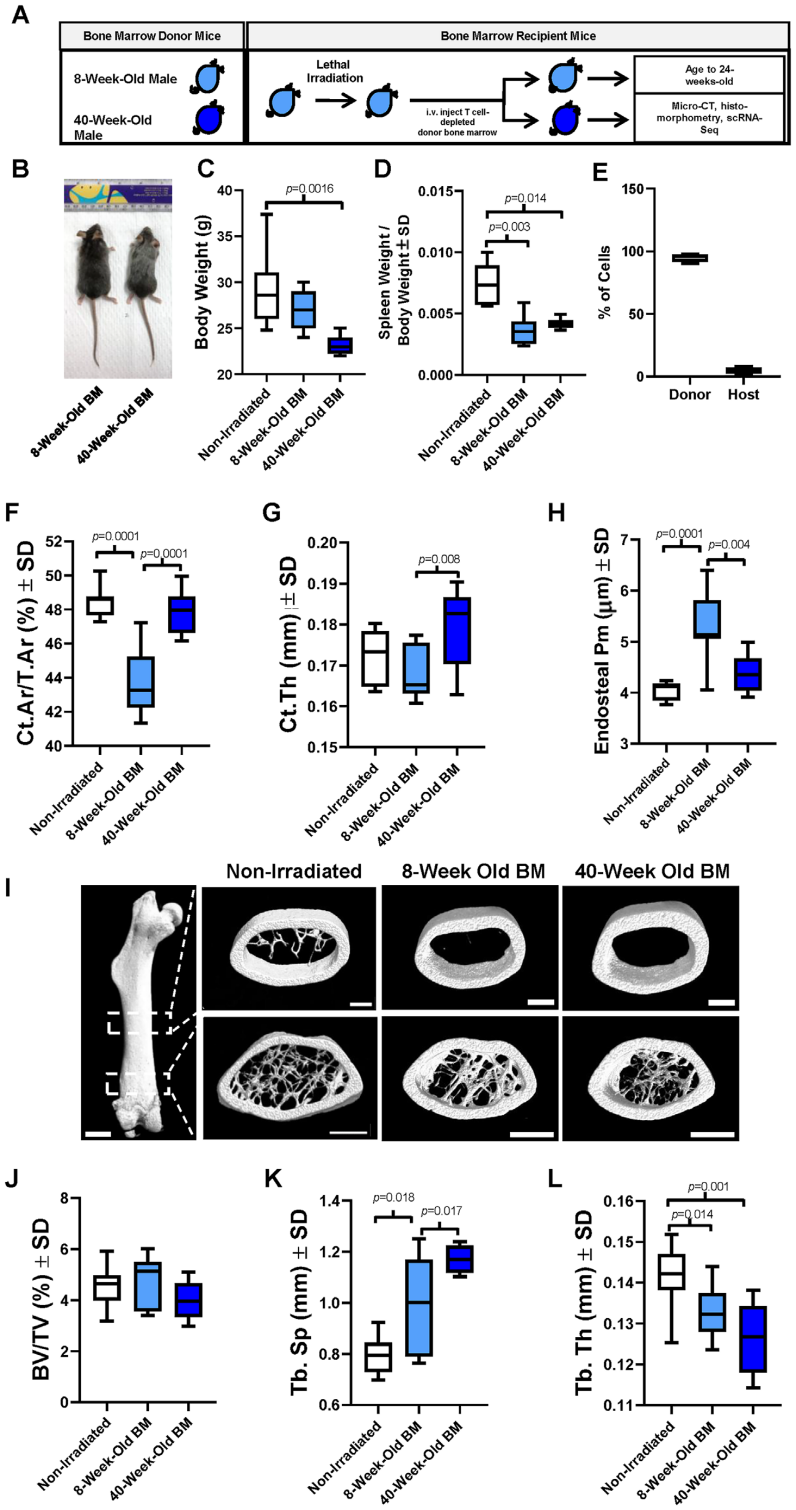
Transplantation of middle‐aged bone marrow enhances cortical bone. (A‐E) Young male recipient mice were lethally irradiated and reconstituted with T cell‐depleted bone marrow (BM) from (1) age‐matched males or (2) 40‐week‐old male mice. Recipient mice were aged to 24 weeks for subsequent analyses. Non‐irradiated (NI) 24‐week‐old males were used for comparisons. (A) Experimental overview. Mice were (B) photographed. (C) Body and (D) spleen weight/body weight was assessed. (E) Percent chimerism via CD45.2^+^/CD45.1^+^ percentage of cells was assessed. Micro‐CT of the femoral midshaft was performed to determine (F) cortical area/total area, (G) cortical thickness, (H) endosteal perimeter (Pm) and (I) reconstructions were generated. The distal femoral region was also assessed to determine trabecular (J) BV/TV, (K) trabecular separation, and (L) trabecular thickness. *p* values less than 0.05 are shown.

EMPs serve as progenitors for osteoclasts that help to pattern the skeletal elements during development. EMPs also expand during intensive hematopoietic regeneration to facilitate blood production. We hypothesized that reactivation of this developmental program may help to maintain bone mass during midlife. To test this hypothesis, we lethally irradiated 6–8‐week‐old male mice and then transplanted bone marrow cells derived from either age‐matched or 40‐week‐old mice (Figure 1A). We based our choice of host age on data demonstrating that Csf1r^+^ yolk‐sac‐derived EMPs are mostly absent by 8 weeks of age [2]. Reconstitution with 40‐week‐old bone marrow led to gross signs of aging, including grey coat colour (Figure 1B). When compared with non‐irradiated controls, mice reconstituted with 40‐week‐old bone marrow showed diminished body weight, whereas mice receiving age‐matched bone marrow did not exhibit changes in body weight (Figure 1C). Splenomegaly can indicate deficiencies in bone marrow reconstitution. While spleen weight per body weight was reduced in mice transplanted with either age‐matched or 40‐week‐old bone marrow, no splenomegaly was observed (Figure 1D). We next evaluated percent chimerism within each group. Within CD45.1 hosts, 98% of bone marrow cells were CD45.2^+^ (Figure 1E), indicating nearly complete chimerism.

We next evaluated how bone marrow age affected bone mass. When comparing animals reconstituted with age‐matched bone marrow to non‐irradiated controls, we observed a 10% reduction in cortical area per total area (Figure 1F,I). In contrast, transplantation of 40‐week‐old bone marrow did not alter cortical bone mass when compared with non‐irradiated controls (Figure 1F,I). Likewise, chimeric mice receiving 40‐week‐old bone marrow demonstrated a 9% increase in cortical bone mass when compared with those receiving age‐matched bone marrow (Figure 1F,I). We did not observe a change in cortical thickness when comparing groups receiving age‐matched or 40‐week‐old bone marrow to non‐irradiated controls (Figure 1G,I). In contrast, transplantation of 40‐week‐old bone marrow increased cortical thickness when compared with mice receiving age‐matched bone marrow (Figure 1G,I). Given the changes in cortical thickness, we also examined the endosteal perimeter. When compared with non‐irradiated controls, delivery of age‐matched bone marrow increased endosteal perimeter, whereas delivery of 40‐week‐old bone marrow did not (Figure 1H,I). Endosteal perimeter was also diminished when comparing mice transplanted with 40‐week‐old bone marrow to those receiving age‐matched bone marrow (Figure 1H,I). Periosteal perimeter was not significantly altered (Figure S1A), but total area and cortical area were impacted when comparing transplanted with either 8‐ or 40‐week‐old bone marrow (Figure S1B,C). We did not observe any changes in distal femoral trabecular bone volume per total volume (Figure 1I,J). In contrast, we did note micro‐architectural trabecular changes. Mice transplanted with either 8‐ or 40‐week‐old bone marrow exhibited increased trabecular separation when compared with non‐irradiated controls (Figure 1I,K). Trabecular separation was also increased when comparing mice reconstituted with 40‐week‐old bone marrow with those receiving age‐matched bone marrow (Figure 1I,K). Trabecular thickness was also diminished within chimeric mice transplanted with either 8‐ or 40‐week‐old bone marrow when compared with non‐irradiated controls, but no differences were observed between chimeric mice (Figure 1I,L). Bone histomorphometry of the trabecular compartment did not support changes in osteoblast number, but we did observe decreased osteoclast number within this region (Figure S2). These results suggest that transplantation of young animals with middle‐aged bone marrow led to increased cortical bone parameters and altered trabecular bone microarchitecture.

**FIGURE 2:**
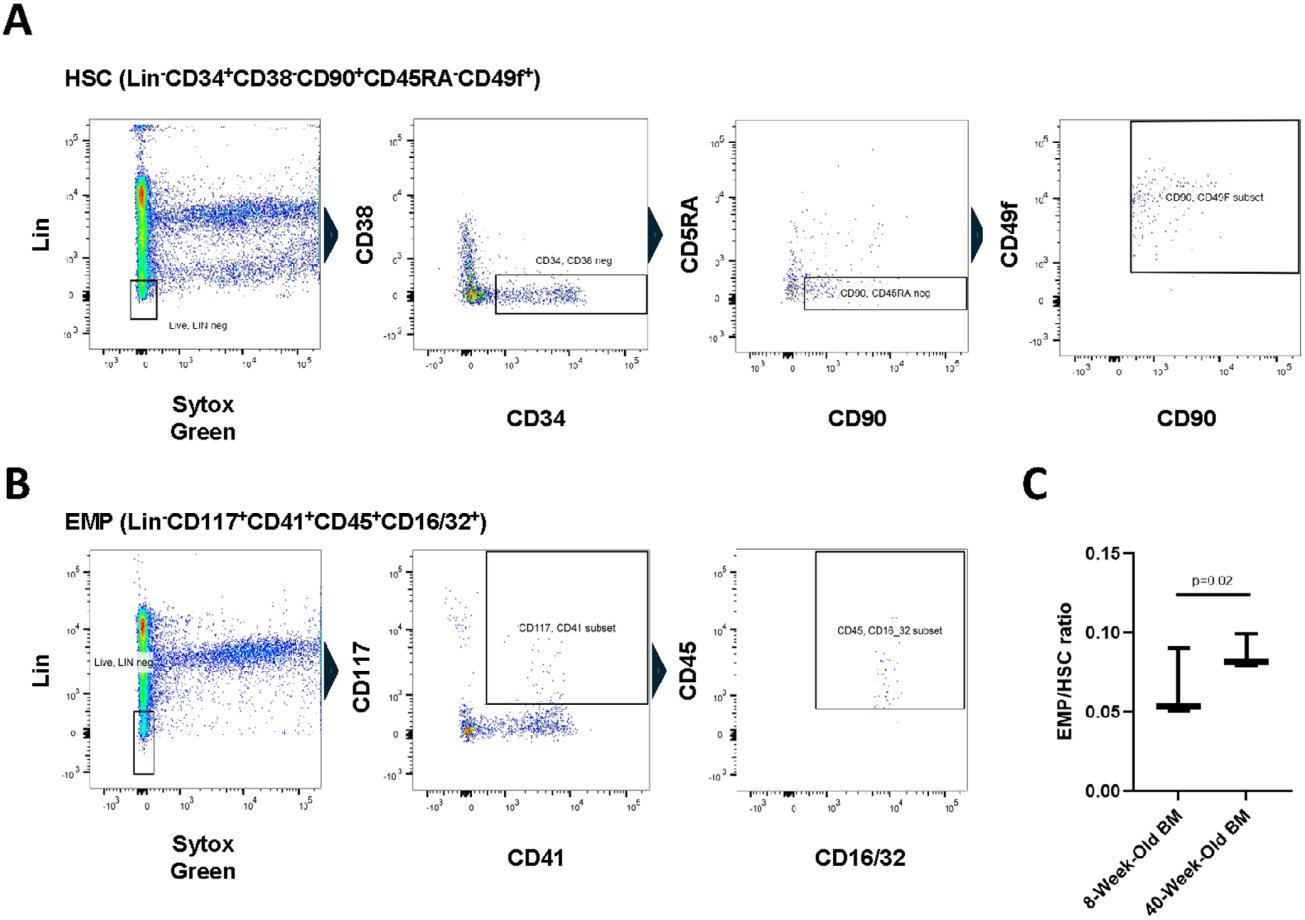
Transplantation of middle‐aged bone marrow enhances the EMP/HSC ratio. Bone marrow was collected from chimeric mice and flow cytometry for (A) Lin^−^CD34^+^CD38^−^CD90^+^CD45RA^−^CD49F^+^ and (B) Lin^−^CD117^+^CD41^+^CD45^+^CD16/32^+^ cells. The ratio of erythro‐myeloid progenitors (EMPs) to hematopoietic stem cells (HSCs) was evaluated.

Enhancement of cortical bone parameters following transplantation of 40‐week‐old bone marrow when compared with age‐matched bone marrow represented a surprising finding. We hypothesized that relative EMP expansion associated with intensive bone marrow regeneration may act to compensate for diminished HSC activity associated with increased age [25] and that this expansion of EMPs may rejuvenate the skeleton. We lethally irradiated 8‐week‐old male hosts and transplanted either 8‐ or 40‐week‐old T‐cell depleted bone marrow. Sixteen weeks after transplantation, we collected the bone marrow and assessed numbers of HSCs (Lin^−^CD34^+^CD38^−^CD90^+^CD45RA^−^CD49F^+^ cells, Figure 2A) and EMPs (Lin‐CD117^+^CD41^+^CD45^+^CD16/32^+^ cells, Figure 2B) by flow cytometry. Next, we determined the relative percentage of EMPs to HSCs. Mice reconstituted with 40‐week‐old bone marrow exhibited a greater percentage of EMPs relative to HSCs when compared with those receiving 8‐week‐old bone marrow (Figure 2C). These results demonstrate that relative expansion of EMPs and/or their cellular derivatives associated with enhancement of cortical bone.

**FIGURE 3:**
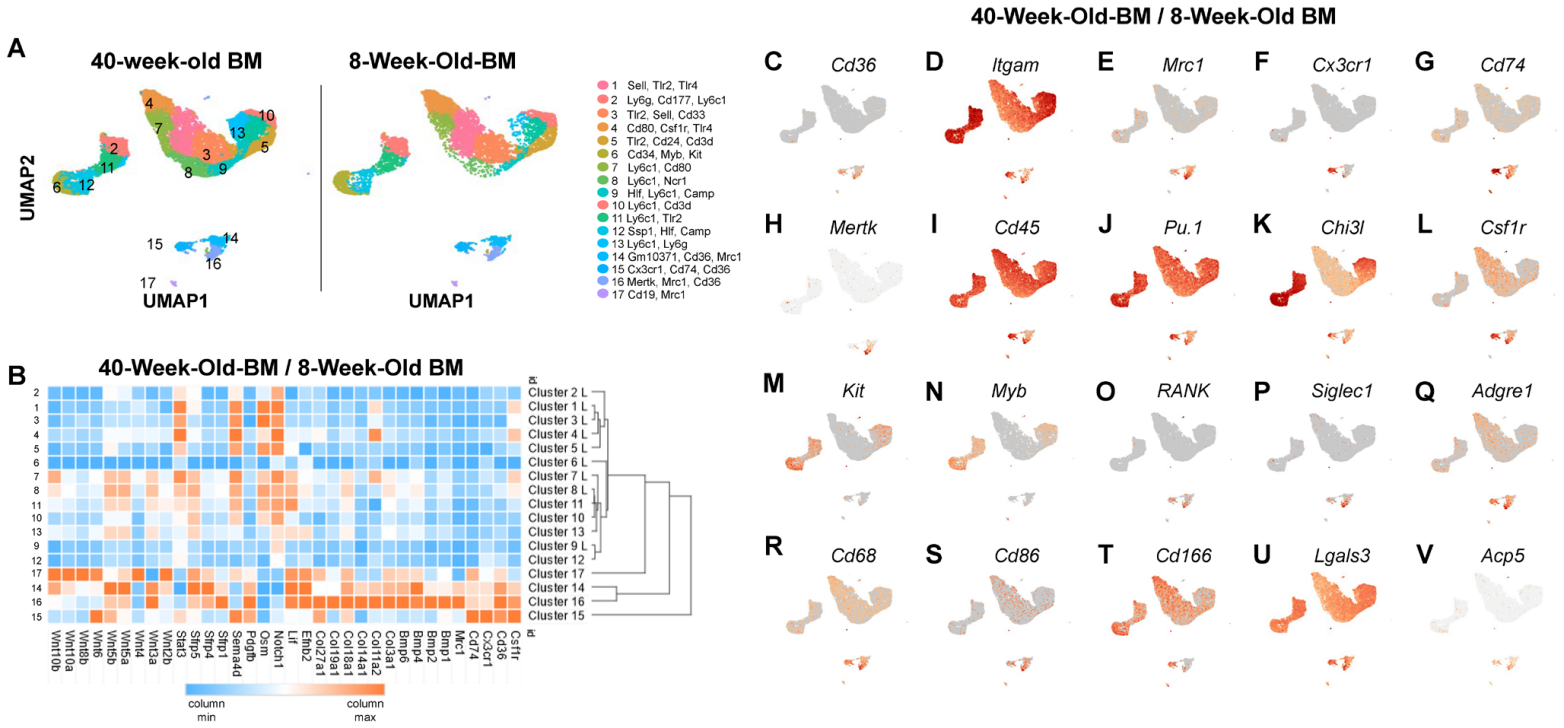
Identification of anabolic bone marrow subsets. (A‐V) Young male recipient mice were lethally irradiated and reconstituted with T cell‐depleted bone marrow (BM) from (1) age matched males or (2) 40‐week‐old male donor mice. Recipient mice were aged to 24 weeks for subsequent analyses. CD11B^+^ cells were collected from mice and scRNA‐Seq was performed using 10× Genomics technology. Resulting data were integrated and comparative analyses were performed. (A) UMAP 2D representation of CD11B^+^ clusters derived from recipient mice transplanted with 40‐week‐old and age‐matched bone marrow. (B) Heatmap of top differentially expressed genes from mice in clusters 14–16 across all clusters. (C‐V) Expression of surface markers used to differentiate clusters.

**FIGURE 4:**
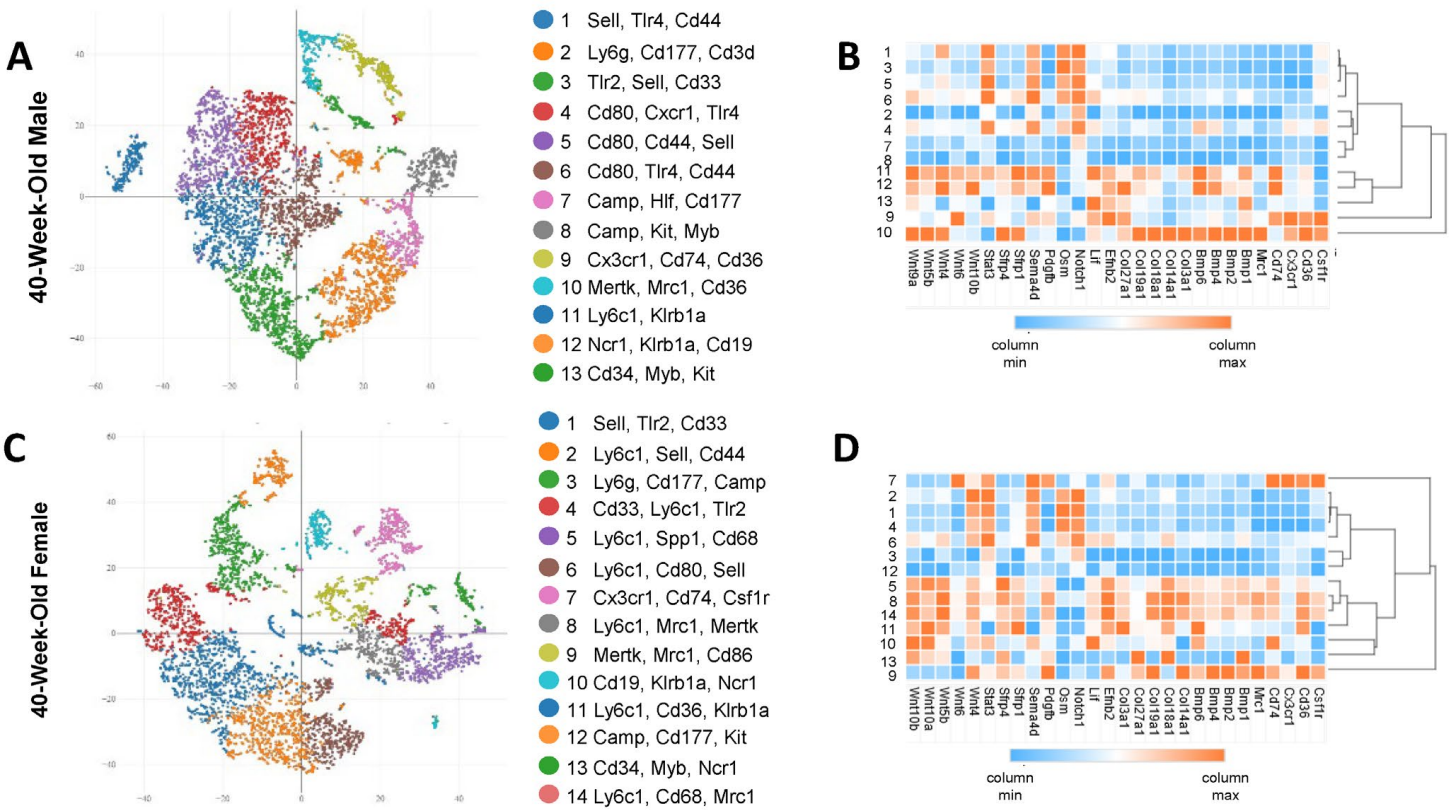
Single cell RNA‐Seq of CD11B^+^ cells from male and female bone marrow reveals subsets of aged myeloid cells that express anabolic cytokines. (A‐D) CD11B^+^ cells from the bone marrow of 40‐week‐old male and female mice were collected and single cell RNA‐Seq was performed. (A) tSNE representation of data obtained from 40‐week‐old males. (B) Heatmap and hierarchical clustering of differentially expressed genes from clusters yielded from 40‐week‐old male data. (C) tSNE representation of data obtained from 40‐week‐old females. (D) Heatmap and hierarchical clustering of differentially expressed genes from clusters yielded from 40‐week‐old female data.

Myeloid lineage cells, along with granulocytes, natural killer cells, and subsets of memory B cells, express the surface marker CD11B. Given the relative expansion of EMPs, we isolated CD11B^+^ cells to determine potential cellular and/or molecular changes driving enhancement of cortical bone parameters via single‐cell RNA sequencing (Figure 3). We first confirmed expression of CD11B/*Itgam* by cells within our analyses (Figure 3D). Comparative analyses between mice reconstituted with 40‐week‐old to 8‐week‐old bone marrow did not identify any unique cell clusters (Figure 3A). Genetic evidence suggests that cortical bone may be controlled specifically by several signaling pathways/components (e.g., Wnt and Notch signaling) [26]. We next evaluated expression of these and related genes across all clusters and noted high expression of Bmps, Collagens, and Wnts by cells within clusters 14–16, and to some extent cluster 17 (Figure 3B). These clusters were further marked by enriched expression of the cell surface marker *Cd36* (Figure 3B,C). Expression of *Mrc1* further distinguished Cluster 14 (Figure 3B,E) whereas Cluster 15 was marked by enriched expression of *Cx3cr1* and *Cd74* (Figure 4B,F,G) and enhanced expression of *Mertk* and *Mrc1* was evident within Cluster 16 (Figure 3B,E,H).

Enriched expression of *Cxc3r1*, *Mrc1*, and *Mertk* suggested that cells within these clusters were monocyte/macrophage lineage cells; therefore, we assessed expression of myeloid lineage markers across all clusters. All clusters expressed markers for hematopoietic cells, including *Cd45*, *Pu.1*, and *Chi3l* (Figure 3I–K). We also noted multiple *Csf1r* ^+^ clusters, including clusters 1, 4, 7, 8, 14, 15, and 16 (Figure 3B,L). Expression of *Kit* and *Myb* marked highly proliferative cells within clusters 6 and 12, which are likely myeloid progenitor cells (Figure 3M,N). *Mrc1* was mostly restricted to clusters 14–16 (Figure 3B,E). Cells within cluster 16 expressed the highest levels of *Mrc1*, but expression was diminished by cells within cluster 15 (Figure 3B,E). Similarly, expression of *Cx3cr1* was restricted to clusters 14–16, with the highest expression observed by cells within cluster 15 (Figure 3B,F). Some cells within clusters 14–16 also expressed *RANK* (Figure 3O), supporting the potential of these cells to give rise to osteoclasts. We also noted high enrichment of *Cd74*, a putative osteomorph marker [27], *Siglec1/Cd169*, and *Cd36* within clusters 14–16 (Figure 3C,G,P). Macrophage markers *Adgre1*, *Cd68*, and *Cd86* were likewise expressed by cells within clusters 1, 4, 7, 8, and 14–16 (Figure 3Q–S). In contrast to recent reports, the putative osteomac markers *Cd166* and *Lgals3* [28] were broadly expressed and not unique to clusters 14–15 (Figure 3T,U). While clusters 14–16 demonstrate expression of *Adgre1* (e.g., F4/80), these clusters expressed the highest levels of *Lgals3*/Mac2. Overall, these data suggest that cells within clusters 14–16 expressed bone anabolic genes and were marked by unique expression of the cell surface marker *Cd36*.

**FIGURE 5:**
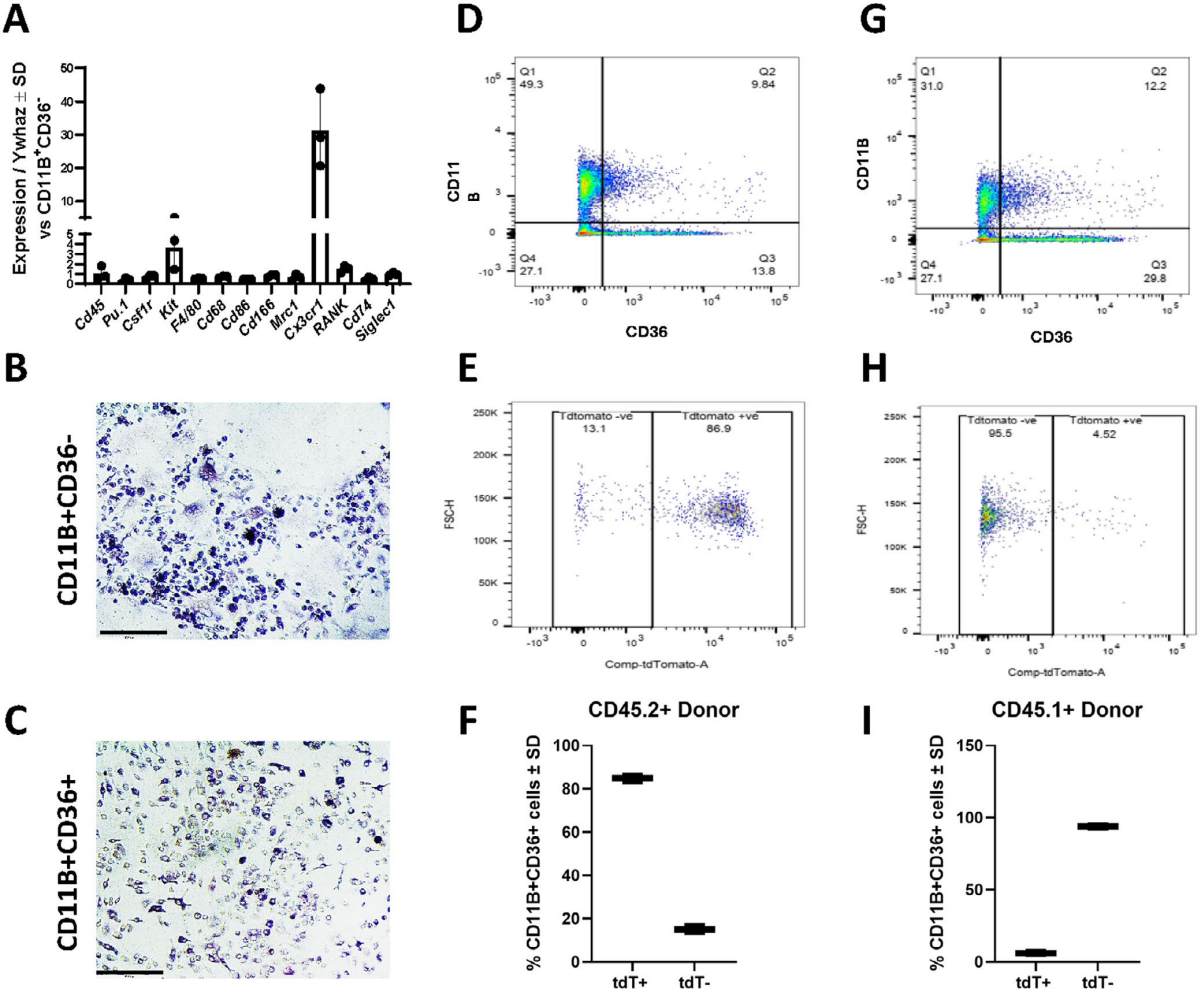
CD11B^+^CD36^+^ cells are of myeloid origin, but do not support osteoclastogenesis. (A‐C) CD11B^+^CD36^−^ and CD11B^+^CD36^+^ cells were isolated from 6–9‐week‐old female mice. (B) Expression of genes was evaluated by qPCR. (B, C) Cells were cultured in osteoclastogenic conditions and TRAP staining was performed. (D‐F) Lethally irradiated CD45.1^+^ mice were transplanted with T cell‐depleted bone marrow from Ai14 tdT:LysM‐Cre1 CD45.2^+^ mice and bone marrow was isolated after 4 weeks. (D) Flow cytometry for CD11B^+^CD36^+^ cells. (E) tdT^+^ cells within the CD11B^+^CD36^+^ population were evaluated and the (F) percent of tdT^+^CD11B^+^CD36^+^ and tdT^−^CD11B^+^CD36^+^ cells was assessed. (G‐I) Lethally irradiated Ai14 tdT:LysM‐Cre1 CD45.2^+^ mice were transplanted with T cell‐depleted bone marrow from CD45.1^+^ mice and bone marrow was isolated after 4 weeks. (F) Flow cytometry for CD11B^+^CD36^+^ cells. (G) tdT^+^ cells within the CD11B^+^CD36^+^ population were evaluated and the (I) percent of tdT^+^CD11B^+^CD36^+^ and tdT^−^CD11B^+^CD36^+^ cells was assessed.

While scRNA‐Seq of CD11B^+^ cells from chimeric mice identified potential cells governing maintenance of cortical bone mass, we wanted to determine if these cells were likewise present under physiological conditions. We therefore profiled CD11B^+^ cells from 40‐week‐old male and female mice (Figure 5). Within males, we noted enriched expression of *Cd36* by clusters 9 and 10 (Figure 4A,B). These clusters were likewise marked by expression of *Cx3cr1* and *Csf1r* (Figure 4A,B). Expression of *Cd74* distinguished cluster 9, whereas *Mrc1* was enriched within cluster 10 (Figure 4A,B). Expression of *Bmp* and *Wnt* genes was highest within cluster 10, but enriched expression was also noted within clusters 11 and 12. When examining female CD11B^+^ cells, we likewise noted enriched expression of *Cd36* within clusters 5, 8, 7, 9, 11 and 14 (Figure 4C,D). These clusters were further distinguished by differential expression of *Cx3cr1*, *Cd74*, and *Mrc1* (Figure 4C,D). Cluster 7 had limited expression of *Wnt* and *Bmp* genes (Figure 4C,D). *Wnt* expression did not characterise cluster 9, whereas cells within this cluster expressed *Bmp* family members (Figure 4C,D). In contrast, clusters 5, 8, and 14 all demonstrated enhanced expression of *Bmp* and *Wnt* genes (Figure 4C,D). These data suggest bone anabolic genes are expressed by CD11B^+^CD36^+^ cells within the bone marrow of 40‐week‐old male and female mice under physiological conditions.

Our scRNA‐Seq data suggested that CD11B^+^CD36^+^ cells were of myeloid origin. We next confirmed enriched expression of myeloid marker genes by CD11B^+^CD36^+^ cells when compared with CD11B^+^CD36^−^ cells. Although all markers were expressed within both populations, we observed enriched expression of *Kit* and *Cx3cr1* (Figure 5A). We likewise determined if these populations supported osteoclastogenesis. Although multinucleated TRAP^+^ cells were observed in cultures derived from CD11B^+^CD36^−^ (Figure 5B), only mononuclear cells were observed using CD11B^+^CD36^+^ cells (Figure 5C).

CD11B and CD36 are both expressed by multiple bone marrow‐resident cells. To establish a myeloid origin of these cells, we performed a modified lineage tracing experiment. We lethally irradiated 8‐week‐old CD45.1^+^ mice and transplanted these mice with T cell‐depleted bone marrow from Ai14 tdT:LysM‐Cre1 CD45.2^+^ mice. In this manner, transplanted bone marrow cells derived from myeloid progenitors were labelled with the Ai14 tdT reporter. Four weeks after transplantation, we collected the bone marrow from chimeric mice and performed flow cytometry for CD11B/CD36 and assessed the percentage of tdT^+^ cells (Figure 5D–F). Of analysed cells, 84.9% of CD11B^+^CD36^+^ cells were also labelled with tdT, suggesting that the vast majority of bone marrow‐resident cells were derived from donor myeloid progenitor cells (Figure 5F). We likewise performed the reciprocal experiment, where Ai14 tdT:LysM‐Cre CD45.2^+^ mice were lethally irradiated and transplanted with unlabelled CDD45.1^+^ T cell‐depleted bone marrow (Figure 5G–I). In this case, 93.75% of CD11B^+^CD36^+^ cells were not labelled with tdT, further supporting that CD11B^+^CD36^+^ cells are of myeloid origins (Figure 5I). Moreover, these data support that CD11B^+^CD36^+^ cells are bone marrow‐resident, donor‐derived cells, and not recruited from the periphery of the host. Additional modified lineage tracing experiments are warranted to pinpoint the developmental origins of these cells.

**FIGURE 6:**
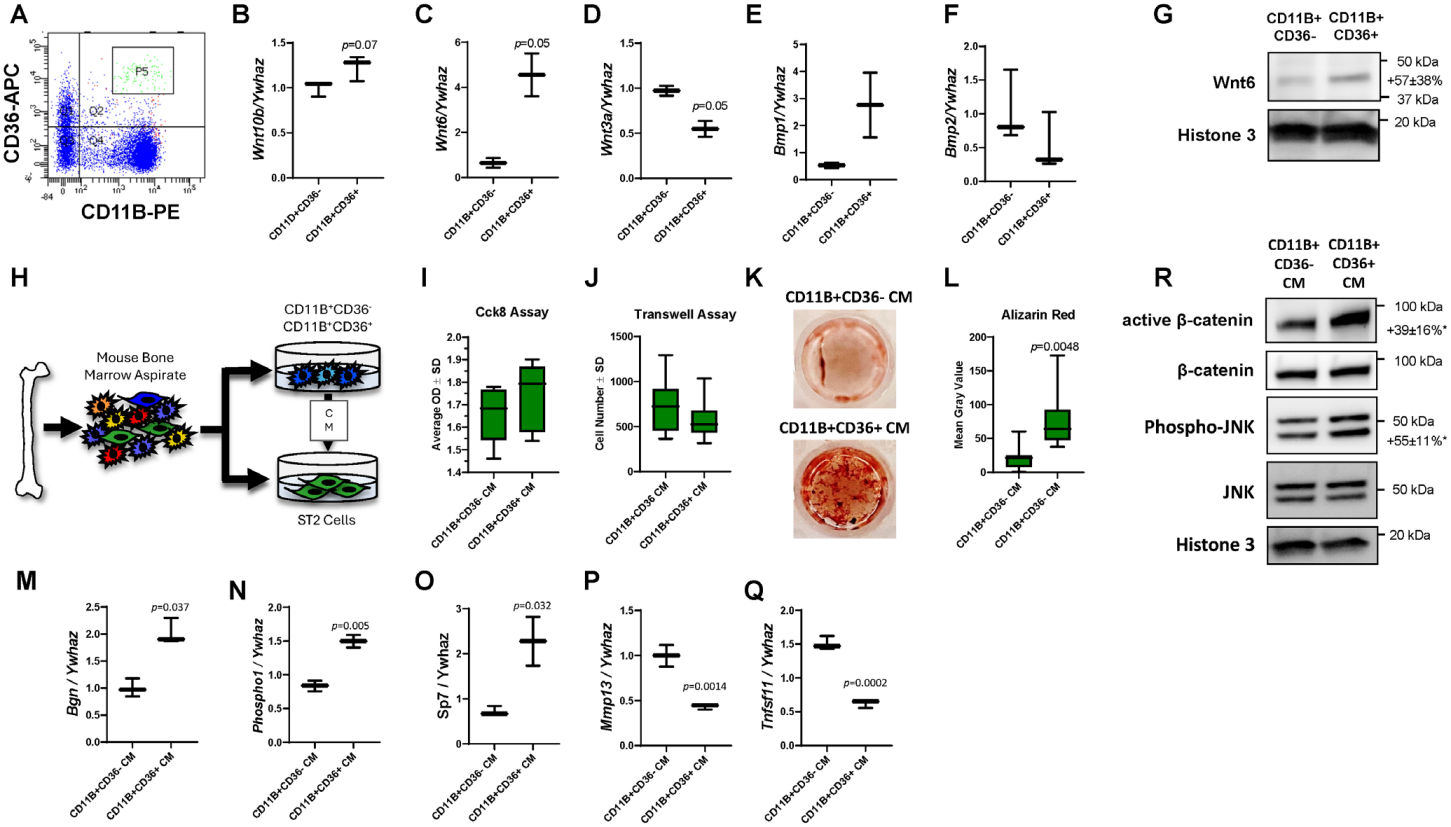
CD11B^+^CD36^+^ Cells Promote Bone Formation. (A) Flow cytometry. CD11B^+^CD36^−^ and CD11B^+^CD36^+^ cells were sorted and gene expression was evaluated by qPCR (B‐F) and western blotting (G), *n* = 3. Fold change ± standard deviations as show to the right the blot, with *p < 0.05 Experimental design (H) for (I‐Q). Osteoprogenitor cells were treated with CM and (I) Cck8 and (J) transwell assays were performed. Osteoprogenitor cells were treated with CM and Alizarin Red staining (K) was performed and quantified (L), n=3. RNA was collected and gene expression (M‐Q) was assessed by qPCR, *n* = 3, *p* values as shown. (R) Osteoprogenitor cells were treated with CM for 60 min and western blotting was performed (*n* = 3) Fold changes ± standard deviations as shown to the right of each blot, with *p < 0.05.

Our scRNA‐Seq analysis revealed that CD11B^+^CD36^+^ cells express several bone anabolic factors, including *Wnt10b*, *Wnt6*, *Wnt3a*, *Bmp1*, and *Bmp2* (Figure 3B). We next examined the percentage of CD11B^+^CD36^+^ cells within the bone marrow. FACS sorting from the bone marrow of 6–9‐week‐old female mice revealed that 8.7% of nucleated bone marrow cells were CD11B^+^CD36^+^ (Figure 6A). We confirmed this when cells were isolated via magnetic‐assisted cell sorting. MACS‐sorted CD11B^+^CD36^+^ cells also exhibited increased expression of *Wnt10b*, *Wnt6*, and *Bmp1* and reduced expression of *Wnt3a* and *Bmp2* when compared with CD11B^+^CD36^−^ cells (Figure 6B–F). *Wnt6* showed the most significant differential expression, which we further confirmed by western blotting (Figure 6G).

Next, we investigated whether factors secreted by CD11B^+^CD36^+^ cells influence osteoprogenitor proliferation, migration, commitment, and mineralization. CD11B^+^CD36^−^ and CD11B^+^CD36^+^ cells were sorted from the bone marrow of 6–9‐week‐old male and female C57Bl/J mice, cultured with M‐CSF for three days, and conditioned medium was collected. To limit potential confounding factors of primary cell cultures (e.g., macrophages within BMSC or calvarial cultures), we utilized the ST2 cell line, an immortalized bipotent osteoprogenitor cell line that can differentiate into adipocytes or osteoblasts. ST2 osteoprogenitor cells were then cultured in conditioned medium to assess proliferation and migration (Figure 6I). Cck‐8 assays indicated no significant changes and transwell assays similarly showed no significant effects on migration (Figure 6J). To evaluate osteoprogenitor commitment and mineralization, ST2 osteoprogenitor cells were cultured in conditioned medium under osteogenic conditions. CD11B^+^CD36^+^ conditioned medium enhanced mineralization (Figure 6K,L), accompanied by increased expression of *Bgn*, *Phospho1*, and *Sp7* (Figure 6M–O). Conversely, we observed diminished *Mmp13* and *Tnfsf11*/RANKL expression by these cultures (Figure 6P,Q). Finally, we assessed the activation of β‐catenin and JNK signalling pathways in ST2 cells cultured in conditioned media from CD11B^+^CD36^−^ and CD11B^+^CD36^+^ cells for one hour. Western blotting revealed elevated levels of active β‐catenin and JNK phosphorylation, indicating increased canonical and/or non‐canonical Wnt signalling (Figure 6R). These results suggest that CD11B^+^CD36^+^ cells produce WNT6, which promotes osteoblast differentiation and bone formation.

**FIGURE 7:**
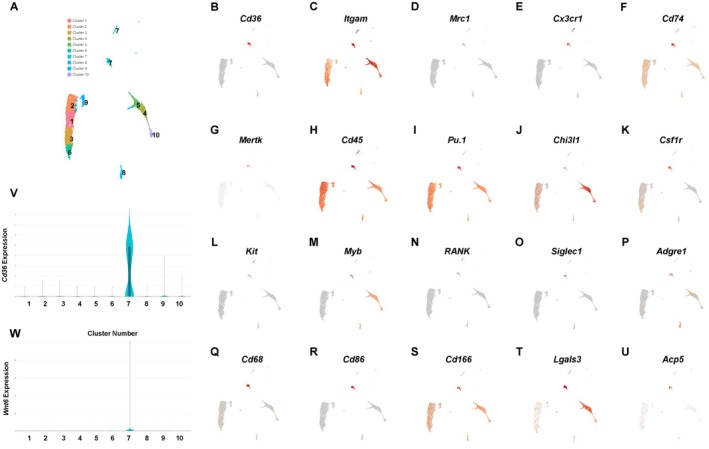
Identification of Human Bone Marrow Macrophage Subsets. CD11B^+^ cells were collected from the bone marrow aspirate of a 45‐year‐old human female and scRNA‐Seq was performed using 10× Genomics technology. (A) UMAP 2D representation of CD11B^+^ clusters. (B–U) Expression of surface markers used to differentiate clusters. Violin plots of CD36 (V) and Wnt6 (W) expression.

To show that our results may translate to humans, we performed single cell RNA sequencing of CD11B^+^ cells from the bone marrow aspirate of a 45‐year‐old human female. Using the pipeline described above, we aligned data to the hg38 reference genome. We identified a cluster of isolated CD11B^+^ cells exquisitely defined by *Cd36* (Figure 7A,B). CD11B^+^ *C* *d* *36* ^+^ cells within cluster 7 also expressed *Cd45*, *Pu.1*, *Csf1r*, *F4/80*, *Cd68*, *Cd86*, *Cd166*, *Cd74*, and *Cx3cr1*, but were mostly negative for *Chi31l*, *Mrc1*, *RANK*, and *Siglec1/Cd169* (Figure 7B–U). We also noted that expression of *Cd74* and *Cd166*, the putative osteomac marker, was broadly expressed by CD11B^+^ cells (Figure 7F,S). Expression of *Wnt6* was likewise restricted to CD11B^+^ cell cluster 7. These results demonstrate that Wnt6 expression is uniquely expressed by CD11B^+^CD36^+^ bone marrow resident cells of middle‐aged women.

## 4 Discussion

The goal of our study was to identify cellular and molecular events that characterize changes to the skeleton during mid‐life. Previous work documented that altered mesenchymal cell function facilitates age‐associated changes to trabecular bone, with consequent myeloid cell skewing [29, 30]. Our study supports this finding, as we observed alterations to trabecular bone microarchitecture. In contrast, our data support that age alters bone marrow composition following intensive hematopoietic regeneration as previously described [10], and those alterations in bone marrow composition facilitate gains in cortical bone. Moreover, our data suggest that a subpopulation of macrophages limit endocortical bone resorption. This may in part explain differences in the onset of cortical bone loss that occurs much later in life when compared with trabecular bone.

Bone resorbing osteoclasts can arise from HSCs or from long‐lived EMPs within the bone marrow. Osteoclasts have an estimated 6‐month half‐life in mice [1, 3, 31]. Evidence suggests that osteoclasts derived from different subsets of progenitors may facilitate bone remodelling in different contexts [2, 32, 33]. For instance, osteoclast progenitors that contribute to homeostatic bone remodelling differ from those mediating bone loss associated with inflammatory conditions [33]. Osteoclasts that remodel bone in other perturbed states or within different bone compartments may likewise differ. Our data suggest that resorption at endosteal cortical bone surfaces may be distinct and limited by secreted factors produced by CD11B^+^CD36^+^ cells. Moreover, our data suggest that CD11B^+^CD36^+^ cells maintain cortical bone mass during midlife. Delivery or induction of these cells could be harnessed to limit the onset of bone loss or mediate gains in bone mass; therefore, further understanding of the developmental origins and epigenetic profile of these cells is warranted.

In our study, we identified a specific myeloid subset that limits cortical bone loss. CD11B^+^CD36^+^ cells were not distinctly marked by expression of *Lgals3*/Mac2 or *Cd166*, two putative osteomac markers; thus, we are unsure if our identified cellular subset overlaps with the osteomac population. Further localization studies are needed to determine this possibility. In addition to CD11B/CD36 surface markers, osteomorph markers (e.g., *Axl*, *Cadm1*, *Ccr3*, *Cd74*, *Vcam1* [27]) were significantly enriched within our identified myeloid subset. Osteomorphs have been speculated to function during the reversal phase of bone remodelling. Indeed, our scRNA‐Seq analyses demonstrated expression of RANK within these clusters, but we were unable to generate osteoclasts from sorted CD11B^+^CD36^+^ cells in vitro. In contrast, CD11B^+^CD36^−^ cells efficiently formed osteoclasts in vitro. This observation could be an artifact associated with antibody‐mediated cell sorting. These data suggest that our identified myeloid subset is potentially distinct from osteomorphs, but we do not know if CD11B^+^CD36^+^ cells could fuse with existing osteoclasts in vivo.

CD36 is a cell surface marker delineating our population of interest, but it is unclear if CD36 is merely a marker for this myeloid subset, or if CD36 plays a functional role in controlling cortical bone mass. CD36 is a membrane glycoprotein that functions as a scavenger receptor for various ligands, including collagen, thrombospondin‐1, anionic phospholipids, and long chain fatty acids. While CD36 is broadly expressed, adipocytes, cardiac myocytes, and immune cells including myeloid cell types (e.g., monocytes, macrophages) exhibit biased expression. CD36 mediates the formation of giant cells, but does not facilitate osteoclast fusion [34]. Our data further confirm that CD11B^+^CD36^+^ cells do not form osteoclasts.

Global deletion of *Cd36* (i.e., *Cd36* ^*−/−*^) resulted in conflicting effects on bone mass. In the first published study, male and female 4‐ to 24‐week‐old *Cd36* ^*−/−*^ mice demonstrated reduced vertebral and femoral cortical bone mass characterized by diminished bone formation [35]. No changes in markers of resorption were noted [35]. Furthermore, in vitro osteoblastogenesis from bone marrow stromal cells was diminished, though the impact of myeloid cell *Cd36* deletion in these cultures could be a confounding factor. Conversely, a later study reported increased vertebral and femoral cortical BV/TV in female 12‐week‐old *Cd36* ^*−/−*^ mice, with no detection of CD36 expression by mature osteoclasts or osteoblasts [36]. Given these conflicting results using *Cd36* global knockout mice, an examination of the role of *Cd36* specifically within monocyte/macrophage‐lineage cells during bone remodelling and regeneration is warranted as understanding the role of *Cd36* in bone biology would offer potential therapeutic targets for managing bone‐related diseases and improving skeletal health.

Our scRNA‐Seq data demonstrated enrichment of secreted factors (e.g., Wnts, Bmps) by myeloid cells within our clusters of interest. Coupled with decreased endosteal perimeter, we speculated that CD11B^+^CD36^+^ cells may limit resorption at endosteal surfaces. This is supported by diminished osteoclast numbers within trabecular bone. Others have demonstrated that bone apposition can also occur at the endosteal surface [37]; therefore, CD11B^+^CD36^+^ cells could likewise be promoting bone formation at that surface. Wnt signalling limits osteoclastogenesis [38]. Likewise, canonical Wnt signalling promotes expression of OPG by osteoblasts, leading to diminished osteoclastogenesis [39]. In contrast, little is known about production of Wnt ligands by other cell types involved in bone remodelling and how this impacts cortical bone resorption. In fact, studies suggest that osteoclasts and bone forming osteoblasts may not be present at sites of bone remodelling at the same time [40, 41, 42], particularly at the endosteal surface. Likewise, age disrupts the temporal and spatial processes of resorption and formation. Our studies point to CD11B^+^CD36^+^ cells as targets to limit cortical bone loss. In future studies, we will determine which factors produced by CD11B^+^ CD36^+^ cells limit osteoclastogenesis and bone resorption.

Our study has several limitations. We did not assess possible contributions of splenic cells to our phenotype. Studies have shown that osteoclast progenitors from the spleen can migrate to bone and participate in bone remodelling and repair [2, 3, 32]. When performing our modified lineage tracing experiments, we noted that CD11B^+^CD36^+^ cells were almost completely donor‐derived. Although this supports the notion that transplanted cells are associated with increased bone mass, we cannot completely rule out the contribution of host cells (e.g., cells recruited from the spleen to the periosteum or elsewhere). Although we speculate that CD11B^+^CD36^+^ cells are not osteomorphs based on their inability to support osteoclastogenesis in vitro, we do not know if this is true in vivo. Artifacts during single cell RNA‐sequencing can arise when fragile cells, including myeloid cells, are lysed or adhere to other cell types [43]. Because of this, we confirmed our results through multiple complementary assays.

Overall, we describe a population of cells that are present within middle‐aged preclinical models and humans that have the capacity to support bone formation. Future studies will be aimed at determining the developmental sources of these cells, localization of CD11B^+^CD36^+^ cells within the bone marrow space, function of CD36 within myeloid populations, as well as the role of CD11B^+^CD36^+^ cells during pathological bone loss and bone regeneration.

## Author Contributions

J.K. and I.Y.K. contributed to investigation, writing of the original draft, editing, formal analysis and data curation. E.K.V., J.C.M., and M.J.S. performed investigation, review and editing of the manuscript, formal analysis and data curation. K.C.M. contributed to funding acquisition, reviewing and editing of the manuscript, and supervision. E.W.B. contributed conceptualization, investigation, funding acquisition, writing of the original draft, review and editing, formala.

## Funding

This work was supported by the National Institutes of Health, R21AR084530, HD055887.

## Conflicts of Interest

The authors declare no conflicts of interest.

## Supporting information


**Data S1:** Supporting Information.
**Figure S1:** Supplementary Transplantation of middle‐aged bone marrow enhances cortical bone. (A‐C) Young male recipient mice were lethally irradiated and reconstituted with T cell‐depleted bone marrow (BM) from (1) age‐matched males or (2) 40‐week‐old male mice. Recipient mice were aged to 24‐weeks for subsequent analyses. Micro‐CT of the femoral midshaft was performed to determine (A) periosteal perimeter (Pm), (B) total cortical area, and (C) cortical area. *p* values less than 0.05 are shown.
**Figure S2:** Supplementary Bone Histomorphometry of Chimeric Mice. (A‐C) Young male recipient mice were lethally irradiated and reconstituted with T cell‐depleted bone marrow (BM) from (1) age‐matched males or (2) 40‐week‐old male mice. Recipient mice were aged to 24‐weeks for subsequent analyses. Bone histomorphometry was performed to enumerate (A) osteoblast number per bone perimeter, and (B) osteoclast number per bone perimeter within the trabecular compartment. *p* values less than 0.05 are shown.

## Data Availability

The data that support the findings of this study are available from the corresponding author upon reasonable request.
