# Supplementary material for: Old Blood, Young Bones: Identification of Middle‐Aged Myeloid Cells That Limit Cortical Bone Loss
Source: J Cell Mol Med. 2026 Mar 23;30(6):e71094. doi: 10.1111/jcmm.71094 (PMC13097527; doi:10.1111/jcmm.71094)
Supplement: Supplementary file 1 — Data S1: Supporting Information. Figure S1: Supplementary Transplantation of middle‐aged bone marrow enhances cortical bone. (A‐C) Young male recipient mice were lethally irradiated and reconstituted with T cell‐depleted bone marrow (BM) from (1) age‐matched males or (2) 40‐week‐old male mice. Recipient mice were aged to 24‐weeks for subsequent analyses. Micro‐CT of the femoral midshaft was performed to determine (A) periosteal perimeter (Pm), (B) total cortical area, and (C) cortical area. p values less than 0.05 are shown. Figure S2: Supplementary Bone Histomorphometry of Chimeric Mice. (A‐C) Young male recipient mice were lethally irradiated and reconstituted with T cell‐depleted bone marrow (BM) from (1) age‐matched males or (2) 40‐week‐old male mice. Recipient mice were aged to 24‐weeks for subsequent analyses. Bone histomorphometry was performed to enumerate (A) osteoblast number per bone perimeter, and (B) osteoclast number per bone perimeter within the trabecular compartment. p values less than 0.05 are shown. [file JCMM-30-e71094-s001.docx]

Supplemental Figure 1


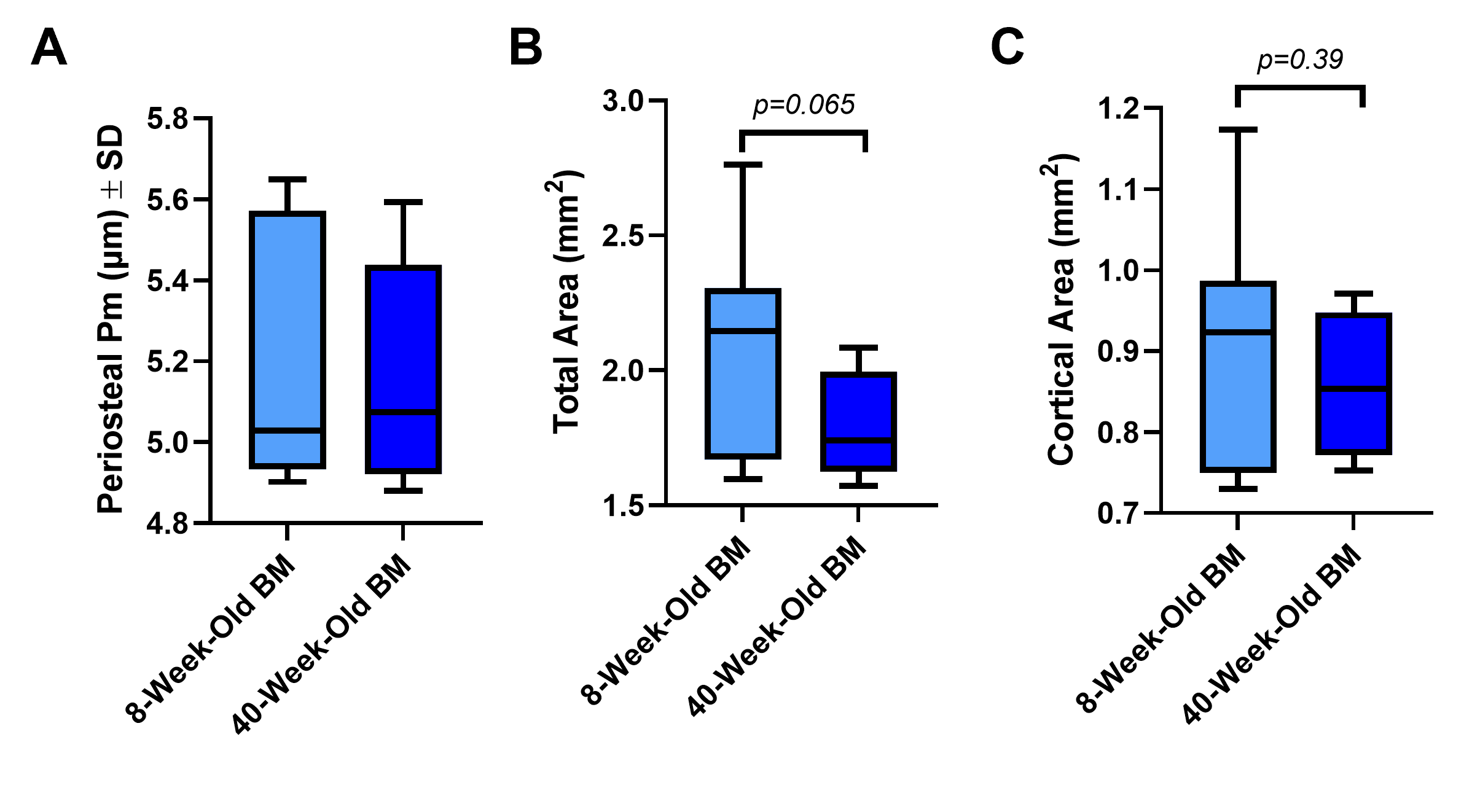


**Supplemental Figure 1.** **Transplantation of middle-aged bone marrow enhances cortical bone.** (A-C) Young male recipient mice were lethally irradiated and reconstituted with T cell-depleted bone marrow (BM) from 1) age-matched males or 2) 40-week-old male mice. Recipient mice were aged to 24-weeks for subsequent analyses. Micro-CT of the femoral midshaft was performed to determine (A) periosteal perimeter (Pm), (B) total cortical area, and (C) cortical area. *p* values less than 0.05 are shown.

Supplemental Figure 2


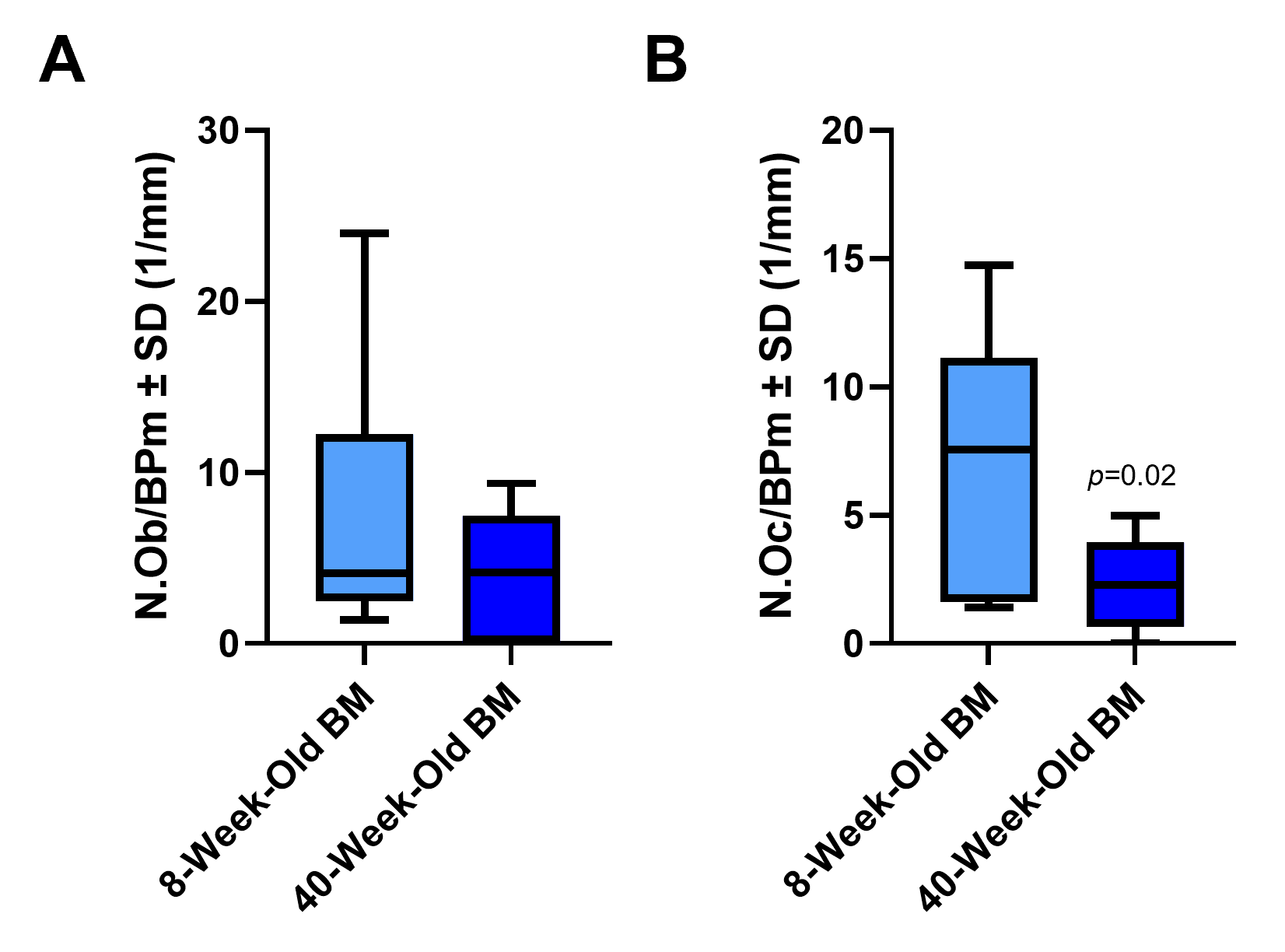


**Supplemental Figure 2. Bone Histomorphometry of Chimeric Mice.** (A-C) Young male recipient mice were lethally irradiated and reconstituted with T cell-depleted bone marrow (BM) from 1) age-matched males or 2) 40-week-old male mice. Recipient mice were aged to 24-weeks for subsequent analyses. Bone histomorphometry was performed to enumerate (A) osteoblast number per bone perimeter, and (B) osteoclast number per bone perimeter within the trabecular compartment. *p* values less than 0.05 are shown.
